# Microbial interactions lead to rapid micro-scale successions on model marine particles

**DOI:** 10.1038/ncomms11965

**Published:** 2016-06-17

**Authors:** Manoshi S. Datta, Elzbieta Sliwerska, Jeff Gore, Martin F. Polz, Otto X. Cordero

**Affiliations:** 1 Computational and Systems Biology Graduate Program, Massachusetts Institute of Technology, Cambridge, Massachusetts 02139, USA; 2 Department of Civil, Environmental and Geomatic Engineering, Institute of Environmental Engineering, ETH Zürich, Zürich 8093, Switzerland; 3 Department of Physics, Physics of Living Systems, Massachusetts Institute of Technology, Cambridge, Massachusetts 02139, USA; 4 Department of Civil and Environmental Engineering, Massachusetts Institute of Technology, Cambridge, Massachusetts 02139, USA; 5 Department of Environmental Systems Science, Institute of Biogeochemistry and Pollutant Dynamics, ETH Zürich, Zürich 8092, Switzerland

## Abstract

In the ocean, organic particles harbour diverse bacterial communities, which collectively digest and recycle essential nutrients. Traits like motility and exo-enzyme production allow individual taxa to colonize and exploit particle resources, but it remains unclear how community dynamics emerge from these individual traits. Here we track the taxon and trait dynamics of bacteria attached to model marine particles and demonstrate that particle-attached communities undergo rapid, reproducible successions driven by ecological interactions. Motile, particle-degrading taxa are selected for during early successional stages. However, this selective pressure is later relaxed when secondary consumers invade, which are unable to use the particle resource but, instead, rely on carbon from primary degraders. This creates a trophic chain that shifts community metabolism away from the particle substrate. These results suggest that primary successions may shape particle-attached bacterial communities in the ocean and that rapid community-wide metabolic shifts could limit rates of marine particle degradation.

## Abstract (web summary)

Particles of organic matter in the ocean harbour microbial communities that digest and recycle essential nutrients. Here, Datta *et al.* use model marine particles to show that the attached bacterial communities undergo rapid, reproducible successions driven by ecological interactions.

## Graphical abstract

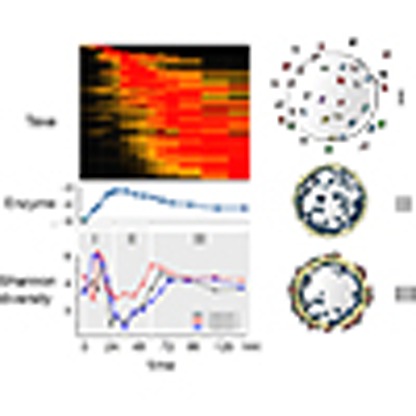

Bacterial colonization of particulate organic matter (POM) in the ocean is a well-known example of microbial community assembly with important implications for global carbon cycling^1^,^2^. On global scales, POM mediates the transfer of nearly two billion tons of carbon from the surface to the deep ocean^3^. However, at micrometre scales, marine particulates serve as spatially isolated, nutrient-rich microhabitats in an otherwise nutrient-poor environment^4^. Microbes from the surrounding seawater, representing a complex colonization pool of bacteria, archaea, eukaryotes and viruses, attach to these particles, eventually forming dense multi-species communities^2^. Within these communities, local interactions between neighbouring cells are predicted to play an important role in shaping community-level structure and function^5^,^6^. These interactions include exploitation of public goods^7^ (for instance, broadcasted degradation products of carbohydrate-active enzymes^8^), antagonistic interactions via antibiotics^9^ and quorum sensing^10^,^11^. Moreover, at regional scales, the efficiency with which bacteria move through a particle ‘landscape' via active or passive dispersal is likely to influence their ecological success^12^,^13^,^14^,^15^,^16^,^17^,^18^. How these processes combine to give rise to dynamics at the level of the community, particularly in the context of a diverse natural microbial assemblage, is still not well understood.

In this work, we investigate the dynamical process by which marine bacteria self-assemble into dense, diverse communities on organic particulates. In the wild, it is often difficult to characterize these community assembly processes and their corresponding drivers, since naturally occurring particles can vary widely in age, size and chemical composition. Here we take an alternative approach, in which we immerse chemically defined, nutrient-rich microparticles in coastal seawater. Using this hybrid approach—maintaining high levels of microbial diversity, while reducing substrate heterogeneity—we track the dynamics of particle colonization with high temporal resolution and explore the underlying drivers of these dynamics. We find that marine bacterial communities assembled on model particles undergo rapid turnover, shifting from a community capable of degrading the particle substrate to one that cannot in a matter of hours. The timescale of this transition could influence the balance between organic matter consumption and biomass build-up in the ocean, potentially a key factor shaping particle remineralization rates in the ocean.

## Results

### Model system

**Figure 1:**
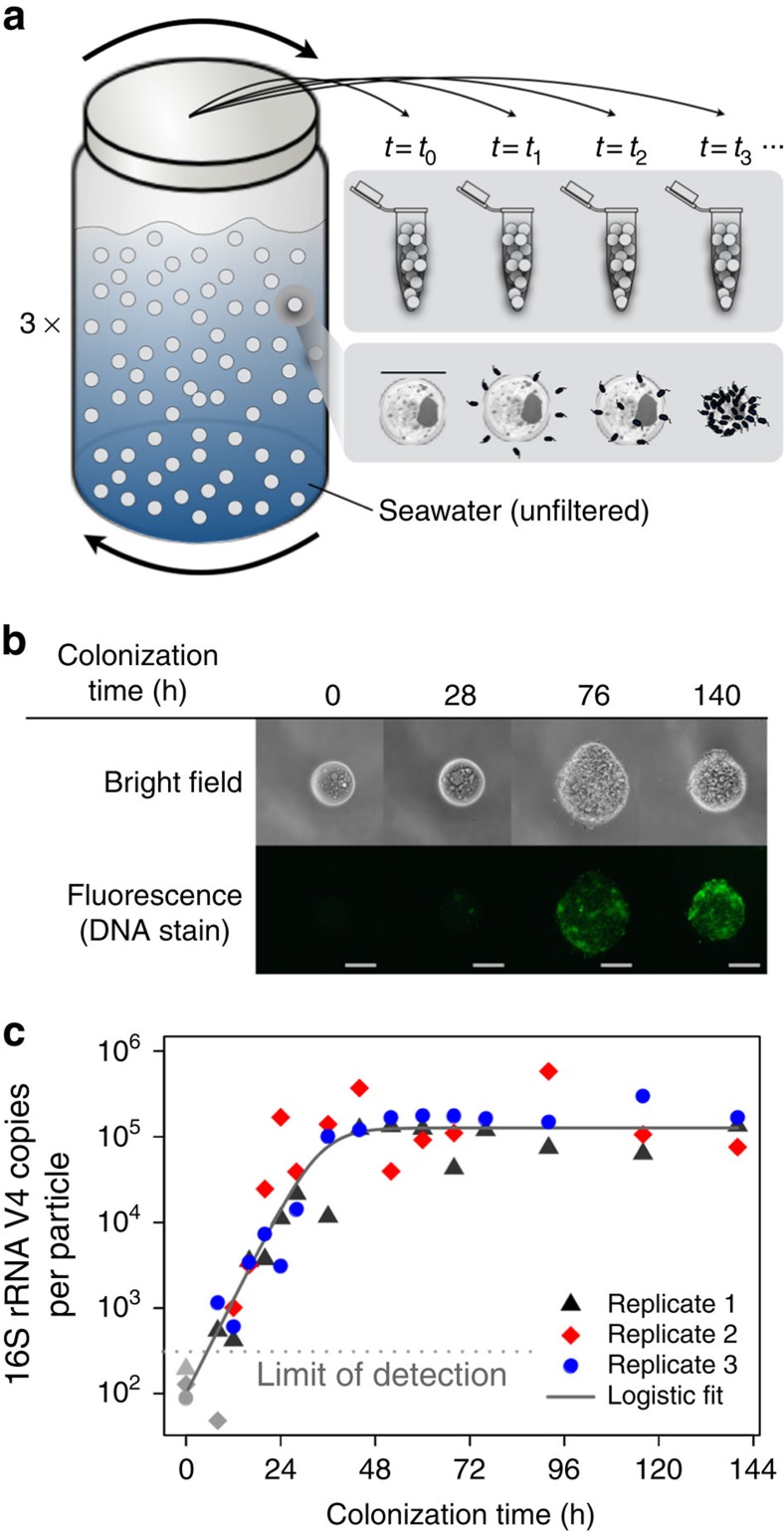
Marine bacteria form communities on model particles. (**a**) Schematic of particle colonization procedure. (**b**) Particle-attached communities stained with SYBR Green I, a double-stranded DNA strain, and imaged with bright field (top) and fluorescence (bottom) microscopy (Supplementary Methods). Scale bars, 25 μm. Note that different particles are depicted for each time point. (**c**) Total 16S rRNA V4 copies per particle over time for three colonization replicates (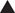, 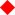, 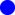). Symbols in grey (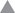, 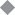, 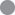) indicate measurements below the limit of detection of the assay. The grey line (-) indicates the fit to a logistic growth model.

To enable studies of microbial community dynamics, we developed a model system inspired by bacterial colonization of POM in the ocean. We simulated POM with paramagnetic micro-particles (Supplementary Fig. 1: median diameter 40.7 μm) made of chitin—a highly abundant biopolymer in the ocean^19^. We incubated these particles in a sample of coastal seawater (Fig. 1a), which contained a diverse microbial assemblage of nearly one million bacteria per millilitre, as well as myriad viruses and small eukaryotes^4^. Over nearly 6 days, bacteria from the surrounding seawater (Supplementary Discussion) self-assembled into communities on the chitin particle microhabitats (Fig. 1b,c; Supplementary Fig. 2). At discrete time intervals, we harvested pools of particles (roughly 1,000 per sample), thus allowing us to reconstruct the average community assembly dynamics occurring over many spatially distinct, but temporally synchronized, particles. To assess the reproducibility of these dynamics, we performed three replicates of the colonization process from a single, well-mixed seawater sample.

### Successions in particle-attached bacterial communities

Despite the extreme diversity of the surrounding microbial assemblage, we found that the overall growth dynamics of particle-attached bacterial communities were surprisingly simple. To characterize these combined growth dynamics, we quantified the number of copies of the V4 hypervariable region of the 16S rRNA gene—a rough proxy for the number of bacteria—present per particle on average over time. Across three colonization replicates, the dynamics were well described by a logistic growth model (Fig. 1c). In particular, bacterial communities initially underwent rapid exponential growth, in which the number of 16S rRNA gene V4 copies doubled every 3.3 h. However, the total abundance saturated at nearly 10^5^ 16S rRNA gene V4 copies per particle after only 40 h of colonization.

**Figure 2:**
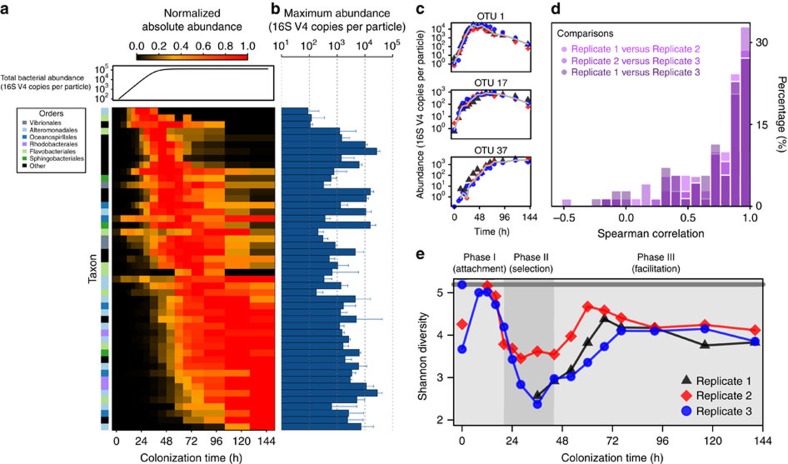
Bacterial communities undergo rapid, highly reproducible successions. (**a**) Absolute abundance trajectories for individual taxa from a single colonization replicate (replicate 2). Individual trajectories are normalized to the maximum value. Colour bar indicates order-level taxonomic identities. Line plot above the heat map shows the logistic fit to the total bacterial abundance trajectory. (**b**) Maximum abundance per particle attained by each taxon. Error bars are s.d.'s (*n*=3). (**c**) Absolute abundance trajectories of three representative taxa across colonization replicates (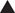, 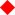, 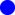). Grey lines indicate the median trajectories. (**d**) Histogram of cross-replicate correlations for individual taxa (Methods). (**e**) Shannon diversity (
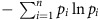
) over time for the three colonization replicates (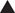, 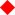, 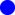). Samples for which sequencing coverage was insufficient for the Shannon diversity to saturate have been omitted. The solid grey line (-) indicates the initial Shannon diversity of the seawater.

Although the total abundance curve saturated early, the underlying colonization dynamics of individual taxa revealed a rapid ecological succession, with wholesale community turnover not only during exponential growth, but also long after the total bacterial abundance had saturated (Fig. 2a). In particular, many taxa experienced a sharp drop in absolute abundance, often by orders of magnitude, soon after reaching their peak absolute abundance levels (for example, operational taxonomic unit 1 (OTU 1) in Fig. 2c). As they dwindled, these taxa were replaced by others, which reached maximum levels that often matched (or exceeded) the earlier colonizers (Fig. 2b), but that declined in turn as still others supplanted them. In total, this dynamic process of community turnover brought 53 highly abundant taxa—present at >1% relative abundance in at least one time point—that each peaked in abundance at times ranging from 16 to 140 h of incubation (Fig. 2a). While microbial successions are widely documented (for example, in the human gut^20^,^21^, the soil^22^ and the marine environment^23^,^24^), such dramatic community turnover has not, to our knowledge, been observed on the spatial (microns) or temporal (hourly) scales documented here.

Given that these dynamics originated from the migration, growth and interactions of many diverse bacteria, we predicted that chance events might give rise to divergent community dynamics, even from the nearly identical starting conditions of our three colonization replicates^25^. However, across these replicates, individual taxon trajectories were highly reproducible (Fig. 2d; Supplementary Fig. 3). For abundant taxa—present at >1% relative abundance at any time point and in any replicate—the median Spearman correlation between individual taxon trajectories from different replicates was >0.8 (Fig. 2d). This high level of reproducibility indicates that technical variation across samples was minimal. However, it also suggests that the average process of community self-assembly is robust to ecological drift, particularly historical contingencies that can arise in a complex microbial milieu.

Importantly, while community turnover occurred continuously, we identified three discrete phases of colonization based upon changes in the community-wide diversity over time (Fig. 2e). In the first phase of colonization (*t*=8–20 h), the communities were at their most diverse (effective number of species, *N*_eff_∼180 OTUs; Supplementary Methods). The second phase was characterized by a significant decline in community-wide diversity, which reached a minimum (*N*_eff_∼20–30 OTUs) after 36–44 h. However, in the third phase, the community-wide diversity rose again, eventually plateauing (*N*_eff_∼50–70 OTUs) after 72 h. Notably, this non-monotonic trend in community diversity held for several diversity metrics (Supplementary Fig. 4).

### Mechanisms underlying successional dynamics

What drives the community shifts that define these three phases of particle colonization? As is often true in plant communities, we hypothesized that temporal changes in the behaviour and metabolism of particle-attached communities may shape the successional patterns that we observed^26^,^27^. To test this hypothesis, we took two complementary approaches. First, we performed metagenomic sequencing of the time series to gain a holistic view of how the metabolic potential of the community changed with time. Second, we amassed and phenotypically characterized a collection of bacterial strains (Supplementary Data 1) isolated from different phases of colonization. Using their 16S rRNA gene sequences, we mapped these isolates to the OTUs originally observed via 16S sequencing (Supplementary Methods; Supplementary Fig. 5). This allowed us to link the phenotypic traits of individual isolates to their taxon's colonization dynamics.

**Figure 3:**
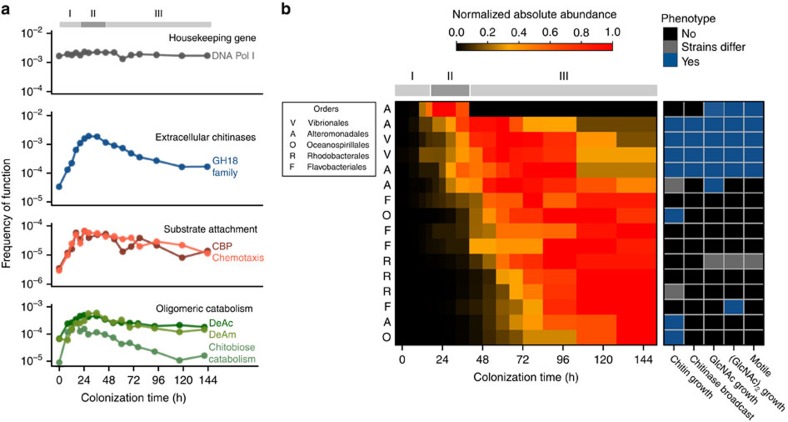
Differences in functional traits between phase II- and phase III-dominant taxa. In both subpanels, phases of colonization (I, II and III) are indicated with the grey colour bar. (**a**) The fraction of read annotations mapped to a given functional category over time. DNA Pol I, DNA polymerase I (EC 2.7.7.7); GH18 family, glycoside hydrolase family 18; CBP, chitin-binding protein (auxiliary activity family 10); Chemotaxis, *N*-acetylglucosamine-regulated methyl-accepting chemotaxis protein; DeAc, *N*-acetylglucosamine-6-phosphate deacetylase (EC 3.5.1.25); DeAm, glucosamine-6-phosphate deaminase (EC 3.5.99.6); Chitobiose catabolism, (GlcNAc)_2_ Catabolic Operon (SEED Subsystem). (**b**) Left heat map: absolute abundance trajectories of isolated taxa. Leftmost letter identifiers show order-level taxonomic identities. Right heat map: whether isolates do (blue) or do not (black) display a functional trait (assays described in Methods; Supplementary Methods). Grey: within-taxon isolates differ in their phenotype. The number of isolates surveyed per taxon ranged from 1 to 3.

Overall, our data suggest that the three phases of colonization were governed by distinct ecological processes: (i) phase I, attachment, (ii) phase II, selection and (iii) phase III, replacement by secondary consumers. In phase I, particle-attached bacterial communities were as diverse as the seawater from which the colonizers originated (Fig. 2e; Supplementary Fig. 4), despite low total bacterial abundance (Fig. 1c). Moreover, the frequencies of gene families associated with chitin metabolism (e.g., GH18 family chitinases) were low (Fig. 3a). This suggests that, at early stages of colonization, the composition of particle-attached communities is not determined by growth on the particle substrate, but instead, may be governed by particle attachment ability. In general, particle attachment is a complex trait influenced by bacteria-particle encounter rates, chemotaxis, biofilm production, and the expression of chitin-binding proteins. Nonetheless, given the diversity of taxa able to colonize particles in phase I, this suggests that particle attachment is a weak selective filter.

By contrast, the dramatic decline in community-wide diversity that defined phase II (*t*=20–44 h) was likely driven by strong ecological selection for chitin metabolism and rapid dispersal ability. In particular, gene families associated with chitin metabolism peaked in relative abundance in phase II (Fig. 3a), while nearly all others remained constant in time (Supplementary Fig. 6). These gene families were associated with multiple stages of chitin metabolism, including extracellular chitin degradation (with GH18 family chitinases), chitin-specific substrate attachment (via chitin-binding proteins), chemotaxis towards chitin monomers, and catabolism of chitin oligomers (Fig. 3a). Indeed, among taxa with isolate representatives, four out of the five taxa that were highly abundant in phase II could grow in culture with chitin as the sole carbon source (Fig. 3b; Supplementary Fig. 7a). Interestingly, all four of these chitin-metabolizing taxa were also able to (i) broadcast extracellular chitinases into the surrounding environment (Fig. 3b; Supplementary Fig. 7e) and (ii) to consume two common chitinase degradation products, chitin monomers (*N*-acetylglucosamine or GlcNAc) and dimers (*N*′,*N*′-diacetylchitobiose or (GlcNAc)_2_) (Fig. 3b; Supplementary Fig. 7b,c). Moreover, all taxa that gained prominence in phase II were motile under laboratory conditions (Fig. 3b; Supplementary Fig. 7d), highlighting that rapid dispersal via active swimming may influence colonization order.

As particle-attached communities entered phase III (*t*=44–140 h), the community-wide diversity rose again from its phase II minimum as, simultaneously, the ecological selection for chitin metabolism and rapid dispersal that defined phase II was relaxed. Community wide, the relative levels of gene families associated with chitin metabolism and chemotaxis towards chitin degradation products declined in phase III, sometimes by orders of magnitude (for example, GH18 chitinases, Fig. 3a). Similarly, among taxa with isolate representatives that reached prominence in phase III, none were motile under laboratory conditions (Fig. 3b), and the majority (8 out of 11) were unable to grow in culture with chitin as the sole carbon source (Fig. 3b; Supplementary Fig. 7a). Incidentally, the minority that could metabolize chitin did not broadcast extracellular chitinases, nor could they typically consume chitin monomers or dimers (Fig. 3b; Supplementary Fig. 7b,c), suggesting a non-canonical mechanism of chitin metabolism compared with their phase II counterparts (Supplementary Discussion). Altogether, despite their widespread inability to consume chitin, the primary particle resource, phase III-dominant taxa often grew to the levels that rivalled those from phase II (Fig. 2b). Thus, phase III marked a community-wide shift in metabolism away from chitin towards other nutrient sources.

**Figure 4:**
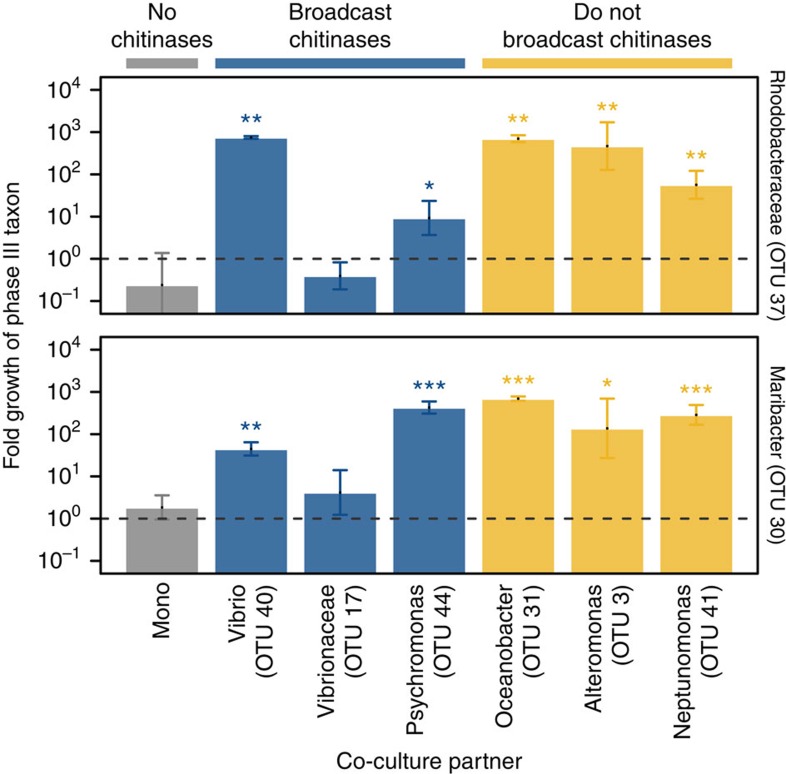
Phase III-dominant taxa that cannot grow on chitin alone grow in co-culture with chitin degraders. Fold growth of two phase III-dominant taxa (OTUs 37 and 30) in monoculture (‘Mono', grey) and in co-culture with chitin-degrading partners (‘OTU X', blue/yellow). Blue bars: partners that broadcast extracellular chitinases. Yellow bars: partners that do not broadcast extracellular chitinases. Strains and their co-culture partners were characterized taxonomically with the Ribosomal Protein Database (RDP) classifier. The lowest level of classification with >80% confidence is indicated for each strain. Asterisks: when fold growth in co-culture is significantly different than in monoculture (two-tailed *t*-test; *P<0.05, **P<0.01, ***P<0.001). Error bars are standard deviations over three biological replicates.

Given their inability to metabolize chitin directly, we hypothesized that phase III-dominant taxa instead consumed nutrient byproducts produced by chitin metabolizers. To test this hypothesis, we co-cultured isolates from two phase III-dominant taxa (from distinct bacterial phyla, Proteobacteria and Bacteroidetes) with each of six chitin metabolizing isolates (representing three orders within Gammaproteobacteria). Of the six chitin metabolizers, three could broadcast extracellular chitinases, while three did not (Figs 3b and 4), suggesting potential differences in their ability to sustain a non-chitin-metabolizing subpopulation^28^. Altogether, we found that isolates of phase III-dominant taxa grew robustly on chitin particles in 10 out of 12 co-cultured pairs, despite their inability to grow in monoculture. Indeed, the enhancement of their growth was often quite dramatic; in some cases, isolates grew 1,000-fold (∼10 doublings) over 7 days in co-culture, with little or no growth in monoculture. Interestingly, the degree of growth enhancement did not depend on whether the chitin metabolizing co-culture partner could broadcast extracellular chitinases (Fig. 4).

How do chitin metabolizers facilitate the growth of phase III-dominant taxa? Previous studies have documented ‘cheater' strains—specialized for consumption of GlcNAc and (GlcNAc)_2_—that do not produce chitinases themselves, but can exploit chitin-degrading taxa by scavenging for their degradation products^19^,^29^,^30^. However, the taxa that dominated phase III were unlikely to be canonical cheaters. In particular, gene families involved in GlcNAc and (GlcNAc)_2_ catabolism decreased in relative abundance from phase II to phase III (Fig. 3a), suggesting that phase III was not enriched in taxa that specialized in the consumption of these products. Similarly, only 1 out of the 11 isolated taxa that were highly abundant in phase III was able to grow in culture with GlcNAc or (GlcNAc)_2_ as the sole carbon source (Fig. 3b; Supplementary Fig. 7b,c). However, in the same minimal medium, these isolates could grow on many other carbon sources (Supplementary Fig. 8), indicating that growth deficits stemmed from a lack of a suitable carbon source, rather than auxotrophies or missing co-factors. More broadly, this implies that chitin metabolizers facilitate the invasion of phase III-dominant taxa by providing them with alternative carbon sources. Possible sources include, but are not limited to, cell debris, biofilm-associated exopolysaccharides, or small metabolic byproducts (for example, organic acids).

## Discussion

Overall, we have demonstrated that bacterial communities colonizing nutrient-rich microhabitats undergo successional dynamics driven by two factors—dispersal limitation and facilitative interactions—that, together, drive primary successions at the scale of tens of microns. Together, our results suggest that the existing theory of successions that has been developed for plants and animals may be applied to complex natural microbial communities, thereby providing a basis for linking microbial community structure to their population dynamics and activity.

Our work also illustrates that micro-scale ecological dynamics may have important consequences for global ecosystem processes. In particular, the rapid successional transition from primary particle degraders (in phase II) to secondary consumers (in phase III) that we observed in our system suggests that the bacteria commonly found on naturally occurring particles may not be the primary particle degraders. Instead, most particle-attached bacteria may be secondary consumers that recycle waste products from primary degraders. These secondary consumers could increase the biomass yield of the particle-attached community, while decreasing particle degradation rate, as they compete with primary degraders for essential resources like space or oxygen. Therefore, the timescale of this transition could influence the balance between organic matter consumption and biomass build-up in the ocean, potentially a key factor shaping particle remineralization rates in the ocean. Further work should be aimed at understanding the impact of particle-attached community dynamics from microscopic to global scales.

## Methods

### Sampling of seawater

Coastal ocean surface water samples were collected on 7 October 2013 from a sampling site located near Northeastern University's Marine Science Center (Canoe Beach, Nahant, MA, USA; 42°25′11.5′′ N, 70°54′26.0′′ W). At the time of sample collection (roughly 15:00 UTC), the water temperature was 16.5 °C, while the ambient air temperature was 18.1 °C. Salinity was measured to be 29.7 p.p.t. using a handheld refractometer (VWR #89370-226).

### Colonization of chitin particles in seawater

Two millilitres of chitin magnetic beads (New England Biolabs #E8036L; roughly 2.5 × 10^5^ beads per ml) stored in 20% ethanol were washed three times with 50 ml of artificial seawater. Beads were resuspended in 100 ml of artificial seawater, resulting in a bead stock at 5,000 beads per ml. In each of three 1-l screw-cap high-density polyethylene bottles, 16 ml of the bead stock were added to 800 ml of unfiltered seawater, yielding a final bead concentration of 100 beads per ml.

Bottles were rotated end-over-end at four rotations per minute on a homemade bottle rotator under ambient lighting and temperature conditions. At each time point and for each bottle sample, 50 ml of the seawater/bead mixture (5,000 beads total) were transferred into a 50-ml conical tube (Corning Life Sciences #352070). Beads were separated from the surrounding seawater with a neodymium magnet (McMaster-Carr #5862K38). The seawater supernatant was transferred back into the original bottle. The beads that remained were then gently washed three times with 50 ml of artificial seawater before being resuspended in 5 ml of artificial seawater. For subsequent analyses (extraction of genomic DNA, isolate collection and bead imaging), 1 ml of washed beads (containing roughly 1,000 beads in total) was transferred into each of five 1.5-ml Eppendorf tubes. At a subset of time points, 1-ml samples were also collected to characterize the surrounding seawater pool.

### Quantification of total particle-attached bacteria over time

As described above, 1,000 beads were prepared from each bottle at each time point. Total bacterial DNA was quantified for each of these samples using a quantitative PCR (qPCR) assay (Supplementary Methods). Briefly, each sample was amplified in a qPCR reaction, allowing a C_t_ value (the number of cycles required for the PCR amplification curve to cross a threshold) to be calculated for all samples. The number of 16S V4 copies present in a sample was calculated from the C_t_ by using a standard curve.

### Illumina 16S library preparation

Genomic DNA was extracted from all samples with the MasterPure DNA Purification Kit (Epicentre #MCD85201; with modifications described in Supplementary Methods). Amplicon libraries (16S rRNA gene V4 hypervariable region) were prepared according to the method described in Preheim *et al.*^31^. Samples were sequenced on an Illumina MiSeq (PE 250+250) at the BioMicro Center (Massachusetts Institute of Technology, Cambridge, MA). Reads were merged and quality filtered with custom scripts, and were clustered into operational taxonomic units (OTUs) (97% identity cutoff) with UCLUST and USEARCH (http://www.drive5.com/usearch/). The relative abundance of each OTU was calculated by normalizing per-OTU read counts by the total number of reads in the sample. The absolute abundance of each OTU at each time point was calculated by multiplying the OTU's relative abundance at a given time point by the total amount of particle-attached bacteria at that time point.

### Plotting absolute abundance trajectories

Only taxa present at a relative abundance >1% at any time point are shown. Plotted values are the medians over three colonization replicates. Data was smoothed with a three-point running median filter and normalized by the maximum. Absolute abundance trajectories are robust to differences in PCR efficiency and inter-taxon 16S rRNA copy number variation (Supplementary Discussion).

### Cross-replicate correlations

For all taxa in a taxon subset—present at >1% relative abundance in any replicate and at any time point—we calculated the Spearman correlation between trajectories in two colonization replicates (for example, replicate 1 versus replicate 2). We repeated this process for all pairs of replicates.

### Metagenomic sequencing of particle-attached communities

For a single replicate time series (replicate 2), metagenomic libraries were prepared for all time points with the Illumina Nextera XT DNA Sample Preparation Kit (Illumina # FC-131-1024) and Illumina Nextera XT DNA Sample Preparation Index Kit (Illumina # FC-131-1001). Sequencing libraries were normalized before sequencing with a modified protocol (Supplementary Methods). All samples were sequenced on an Illumina MiSeq (PE 250+250) at the Genomic Diversity Center (ETH Zürich, Zürich, Switzerland).

### Functional annotation of metagenomic reads

Before annotation, reads were quality filtered using the standard quality-control pipeline in MG-RAST (http://metagenomics.anl.gov/). Briefly, reads are pre-processed by using SolexaQA to trim low-quality regions. Subsequently, artificial duplicate reads were identified (using a k-mer approach) and removed. Remaining sequences were screened for matches to model organisms (for example, human, mouse, cow, fly) and also removed.

Quality-filtered reads were assigned to functional categories using two methods. In the first method (used for ‘DNA Pol I', ‘Chemotaxis', ‘Chitobiose catabolism', ‘DeAc' and ‘DeAm'), reads were annotated in MG-RAST with SEED Subsystems, manually curated, hierarchical functional categorization system. Annotation transfer cutoffs were the defaults (*e*-value<10^−5^; minimal alignment length=60 bp), although, qualitatively, results were consistent over a wide range of values. Note that MG-RAST allows a single read to be assigned to multiple functional categories (that is, number of read annotations≥number of reads). Therefore, within each sample, counts were normalized to the number of annotations, rather than to the number of reads.

The second method (used for ‘GH18 family' and ‘CBP') involved annotation with manually curated HMMs provided by the dbCAN database (http://csbl.bmb.uga.edu/dbCAN/). The database of metagenomic read sequences was searched with profile HMMs for GH18 (chitinases) and AA10 (chitin-binding proteins) using hmmsearch (HMMER 3.1b1, May 2013, http://hmmer.janelia.org/).

### Culturing isolates from particle samples

As described above, tubes containing 1,000 beads were prepared from each incubation bottle at each time point. At a subset of these time points (*t*=8, 24, 52, 76, 92 and 140 h), tubes were sonicated in a bath sonicator (Cole-Parmer #8891, now discontinued) on ‘low' for five cycles (30 s on, 30 s off). Beads were separated from the supernatant with a neodymium magnet (McMaster-Carr #5862K38). The supernatant was then divided and plated at three different dilutions (1:10^4^, 1:10^5^, 1:10^6^) on each of two types of plates: (1) Marine Broth 2216 (Difco #279110) or (2) Tibbles–Rawling minimal media with 0.2% *N*-acetylglucosamine. Both plate types were prepared with 1.5% agar (BD #214010). Plates were incubated at room temperature for 7 days to allow both slow-growing and fast-growing strains to become visible.

Following growth on plates, roughly 50 colonies were picked as representatives from each time point. To purify strains for subsequent analyses, colonies were re-streaked three times onto fresh plates containing the medium from which they were first picked. Stocks of strain isolates were prepared by first growing strains for 2 days at room temperature in Marine Broth 2216 liquid medium (Difco #279110), and then mixing the saturated culture and 80% glycerol in equal volumes in a cryovial. All stocks were stored at −80 °C. For taxonomic classification of isolates, the 16S rRNA gene of each strain was sequenced via Sanger sequencing (Supplementary Methods). This information was used to map isolates to OTUs identified via culture-independent methods (Supplementary Methods).

### Growth experiments with isolates

Media was prepared by supplementing Tibbles–Rawling minimal media with the desired carbon source. Strains were pre-grown to saturation for 48 h in Marine Broth 2216 medium prepared according to the manufacturer's instructions (Marine Broth 2216, dehydrated; Difco #279110). When strains were to be grown on chitin, strains were pre-grown in Marine Broth 2216 medium supplemented with 1,000 chitin beads per ml.

Isolate growth was assessed on each of three carbon sources: chitin resin (New England Biolabs #S6651L; added at 10^3^ beads per ml), *N*-acetylglucosamine (Sigma-Aldrich #A3286-100G; 0.5% w/v), and *N*,*N*′-diacetylchitobiose (D1523-10MG; 0.1% w/v). For most cases, culture growth was quantified by measuring OD600 with a spectrophotometer (Tecan Infinite F500). However, growth on chitin beads proved difficult to measure accurately with standard optical density measurements. Instead, culture growth was estimated based on the change in total DNA content over time. At discrete time points, 500 μl of the culture was transferred into a 1.5-ml tube. Total genomic DNA was extracted from each of these samples using a MasterPure DNA Purification Kit (Epicentre #MCD85201; as described above). Total double-stranded DNA content was quantified for each sample with a Quant-iT PicoGreen dsDNA Assay Kit (Life Technologies #P7589).

### Chitinase broadcasting assay

A plate-based chitin clearing assay was used to assess chitinase broadcasting ability. Bacterial cultures were grown in Marine Broth 2216 until saturation. A small volume of these saturated cultures (typically 5 μl) was spotted onto a chitin clearing assay plate containing colloidal chitin stained with Remazol Brilliant Violet 5R. Colloidal chitin was prepared and stained as previously described^32^, using an aqueous solution of 1.5% w/v sodium dichromate (Sigma #398063-100G) and 1.5% w/v potassium sodium tartrate (Sigma #217255-100G) as a mordant. After cultures were spotted onto plates, plates were incubated at room temperature for 5 days before imaging.

### Motility assay

A standard agar stab assay was used to assess the potential for motility among isolates. Motility test agar medium was prepared with Marine Broth 2216 (Difco #279110), Bacto Agar (BD #214010) (0.25% w/v) and 2,3,5-triphenyltetrazolium chloride solution (Sigma-Aldrich #17779-10X10ML-F) and autoclaved. Media was aliquoted in autoclaved glass tubes (VWR #47729-576) with plastic closures (Cole-Parmer #EW-04500-01; 5 ml of media per tube) and allowed to cool to room temperature. Using an inoculating needle (Thomas #TL0000), a stab inoculation was made from a single colony for each strain into a media-filled glass tube. Tubes were incubated at room temperature for 7 days before cultures were analysed. Evidence of motility was assessed visually. If growth occurred only along the stab line, strains were considered non-motile under these conditions; otherwise, strains were deemed motile. All results were confirmed via microscopy with liquid cultures in Marine Broth 2216 medium.

### Isolate co-culture experiments

To characterize interactions between community members, we performed co-culture experiments with pairs of strain isolates. In each case, a chitin-degrading strain was mixed with a non-chitin-degrading strain, with the degrader in large excess (degrader:non-degrader≈90:10). Combinations of strains, as well as monocultures of each strain, were grown in Tibbles–Rawling minimal media with chitin beads (1,000 beads per ml) at a total starting cell density of 10^6^ cells per ml. Cultures were grown for 7 days at room temperature and rotated end-over-end at six rotations per minute. For each culture, samples were harvested at the beginning (*t*=0 days) and end (*t*=7 days) of the growth period and frozen at −80 °C for subsequent analyses.

Quantifying the total number of cells in each sample is experimentally challenging. Therefore, the total amount of genomic DNA present in each sample was measured. First, total genomic DNA was extracted from each of these samples using a MasterPure DNA Purification Kit (Epicentre #MCD85201; modifications in Supplementary Methods). Then, total double-stranded DNA content was quantified for each sample with a Quant-iT PicoGreen dsDNA Assay Kit (Life Technologies #P7589).

To estimate the relative abundance of each strain within the co-cultures, amplicon libraries (16S rRNA gene V4 hypervariable region) were prepared according to a previously described protocol^31^. Samples were sequenced on an Illumina MiSeq (paired-end, 250-bp reads) at the BioMicro Center (Massachusetts Institute of Technology, Cambridge, MA). The absolute abundance of each strain in each sample was calculated by multiplying the strain's relative abundance in a given sample by the total amount of genomic DNA present in that sample.

### Data availability

Sequence data that support the findings of this study have been deposited in the NCBI databases BioProject (with accession code PRJNA319196) and BioSample (with accession codes SAMN04886652 to SAMN04886699). Isolate 16S sequence and phenotype data can be found in Supplementary Data 1. The authors declare that all other data supporting the findings of this study are available within the article and its Supplementary Information files, or from the corresponding author upon request. Scripts for processing data can be found at https://github.com/mdatta8788/chitinParticlesSuccession. All scripts are also available from the corresponding author upon request.

## Additional information

**How to cite this article:** Datta, M. S. *et al.* Microbial interactions lead to rapid micro-scale successions on model marine particles. *Nat. Commun.* 7:11965 doi: 10.1038/ncomms11965 (2016).

## Supplementary Material

Supplementary Information Supplementary Figures 1-8, Supplementary Discussion, Supplementary Methods and Supplementary References

Supplementary Data Isolate phenotypes.
