# Supplementary material for: Microbial interactions lead to rapid micro-scale successions on model marine particles
Source: Nat Commun. 2016 Jun 17;7:11965. doi: 10.1038/ncomms11965 (PMC4915023; doi:10.1038/ncomms11965)
Supplement: Supplementary Information — Supplementary Figures 1-8, Supplementary Discussion, Supplementary Methods and Supplementary References [file ncomms11965-s1.pdf]

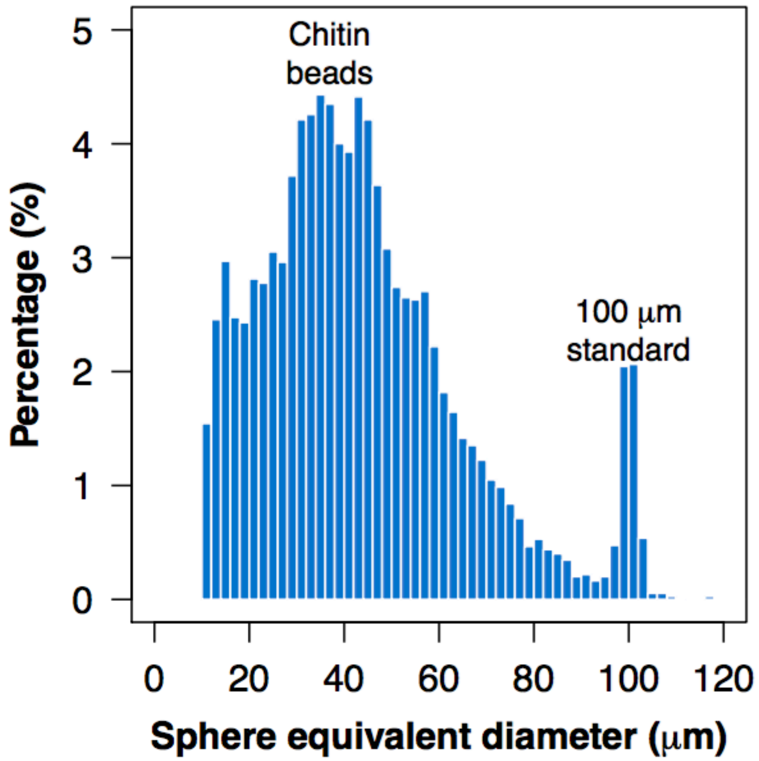

Supplementary Figure 1 | Size distribution of chitin particles. For many individual particles, the sphere equivalent diameter was measured with a Coulter counter (Beckman Coulter Multisizer 4, 560 µm aperture). Note that, for an irregularly shaped object, the sphere equivalent diameter refers to the diameter of a perfectly spherical object of identical volume. The chitin particles that we considered were roughly spherical, but irregularly shaped particles were also observed. As an internal standard, spherical polystyrene particles of a known diameter (100 µm, Thermo Scientific #4310A) were added to the sample before measurement.

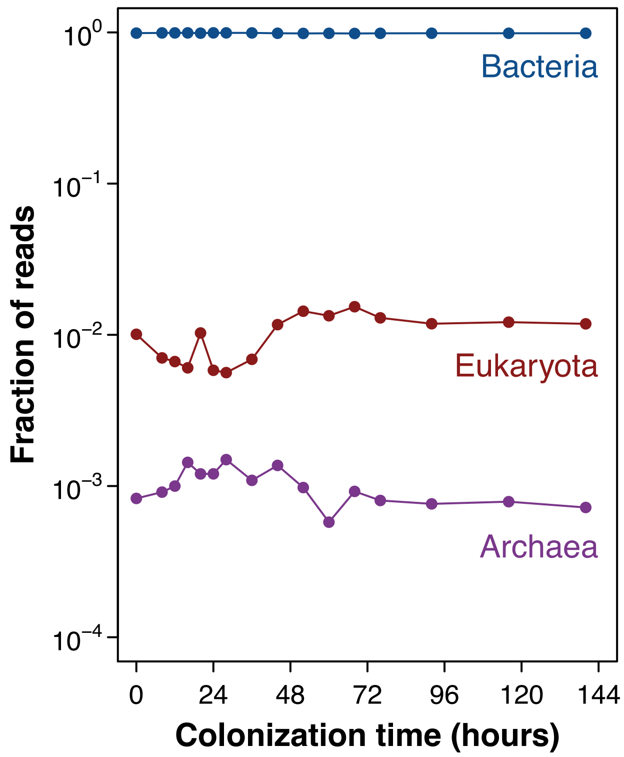

Supplementary Figure 2 | Bacteria are the dominant colonizers on particles. The fraction of read annotations corresponding to each of three taxonomic domains as a function of time. Metagenomic reads were annotated with MG-RAST (BLAT search against the M5NR database, e-value < 10^{-5}; length > 60 basepairs). Note that, in the standard MG-RAST pipeline, a single read may have more than one annotation. Thus, the number of read annotations is larger than the number of reads.

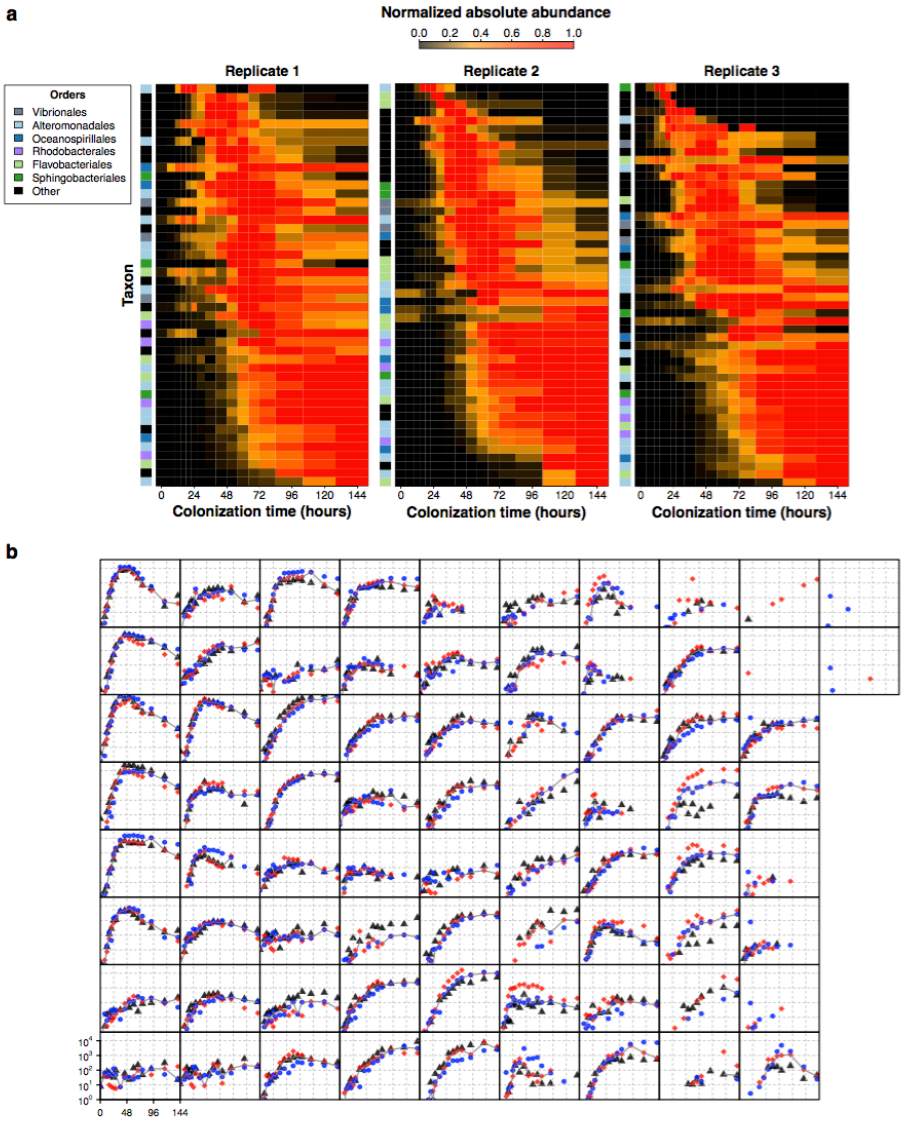

Supplementary Figure 3 | Colonization dynamics of individual bacterial taxa are highly reproducible across replicates. a, Normalized smoothed absolute abundance trajectories for individual taxa in each of three colonization replicates. Each row corresponds to the dynamics of a single taxon (only taxa present at a relative abundance > 1% at any time point in a given replicate are shown). Trajectories were smoothed with a three-point running median filter and normalized by the maximum abundance attached by that taxon such that all values range from 0-1. Taxa are ordered by increasing center of mass of the trajectory. **b,** Absolute abundance trajectories of all taxa present above the relative abundance threshold (1%) in any of the replicates (▲, ◆, ●). Grey lines indicate the median trajectory for a given taxon. All taxon trajectories are plotted with the same x- and y-axis limits.

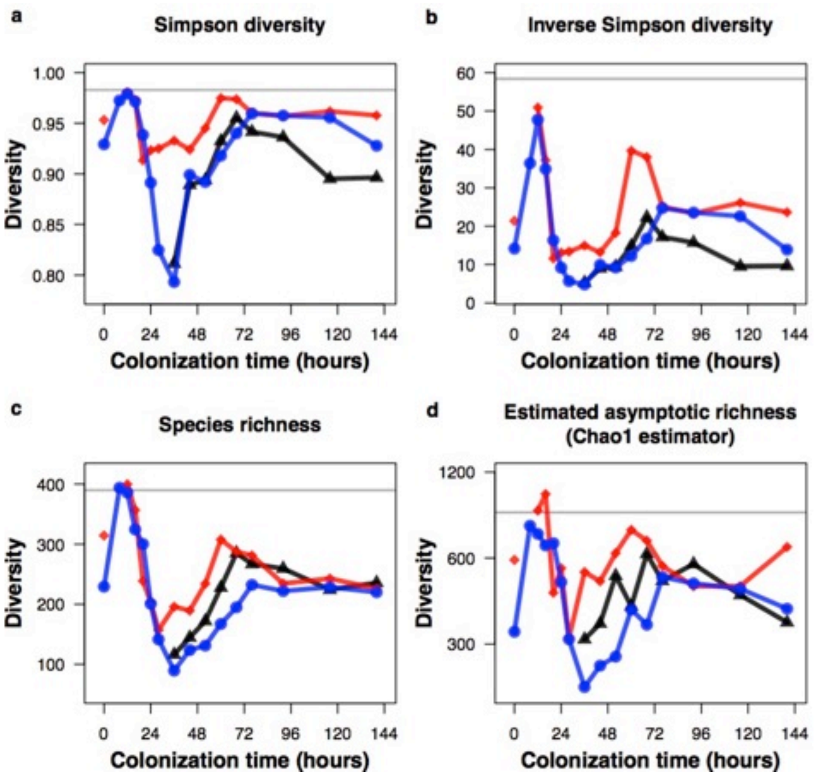

Supplementary Figure 4 | Non-monotonic temporal trend in community diversity holds for other alpha diversity metrics. Particle-attached community diversity over time in each of three replicates (▲, ◆, ●), as calculated with several different diversity metrics. In all plots, the diversity of the initial seawater inoculum (t = 0 hours) is also indicated (—). In all cases, samples with less than 1500 sequenced reads were excluded. **a,** Simpson diversity ($D = 1 - \sum_{i=1}^{n} p_i^2$) at each timepoint. **b,** Inverse Simpson diversity ($D = 1/\sum_{i=1}^{n} p_i^2$) at each timepoint. **c,** Species richness (number of unique OTUs observed) at each timepoint. To account for differences in sampling effort, each community was subsampled to 1,500 reads before calculating species richness. Thus, only the relative species richness is meaningful here. Qualitative differences in species richness are robust to subsample size. **d,** Estimated asymptotic species richness over time. Asymptotic estimators extrapolate the number of species that would be observed, given infinite sampling effort. In particular, the Chao1 estimator is a robust estimator of the minimum asymptotic species richness[31].

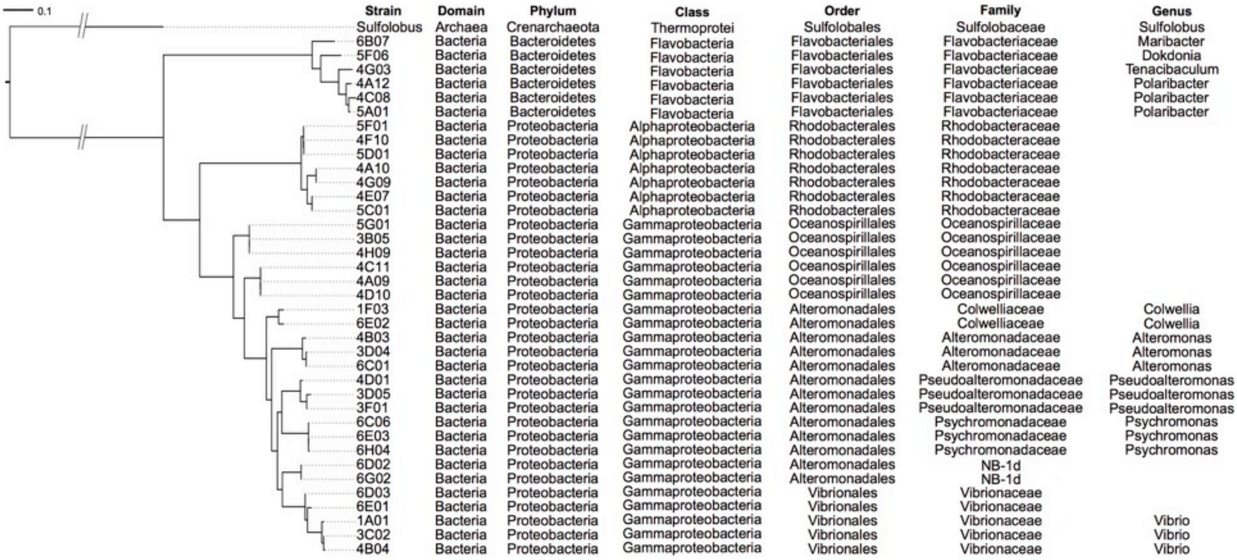

Strain	Domain	Phylum	Class	Order	Family	Genus
Sulfolobus	Archaea	Crenarchaeota	Thermoprotei	Sulfolobales	Sulfolobaceae	Sulfolobus
6B07	Bacteria	Bacteroidetes	Flavobacteria	Flavobacteriales	Flavobacteriaceae	Maribacter
5F06	Bacteria	Bacteroidetes	Flavobacteria	Flavobacteriales	Flavobacteriaceae	Dokdonia
4G03	Bacteria	Bacteroidetes	Flavobacteria	Flavobacteriales	Flavobacteriaceae	Tenacibaculum
4A12	Bacteria	Bacteroidetes	Flavobacteria	Flavobacteriales	Flavobacteriaceae	Polaribacter
4C08	Bacteria	Bacteroidetes	Flavobacteria	Flavobacteriales	Flavobacteriaceae	Polaribacter
5A01	Bacteria	Bacteroidetes	Flavobacteria	Flavobacteriales	Flavobacteriaceae	Polaribacter
5F01	Bacteria	Proteobacteria	Alphaproteobacteria	Rhodobacterales	Rhodobacteraceae	
4F10	Bacteria	Proteobacteria	Alphaproteobacteria	Rhodobacterales	Rhodobacteraceae	
5D01	Bacteria	Proteobacteria	Alphaproteobacteria	Rhodobacterales	Rhodobacteraceae	
4A10	Bacteria	Proteobacteria	Alphaproteobacteria	Rhodobacterales	Rhodobacteraceae	
4G09	Bacteria	Proteobacteria	Alphaproteobacteria	Rhodobacterales	Rhodobacteraceae	
4E07	Bacteria	Proteobacteria	Alphaproteobacteria	Rhodobacterales	Rhodobacteraceae	
5C01	Bacteria	Proteobacteria	Alphaproteobacteria	Rhodobacterales	Rhodobacteraceae	
5G01	Bacteria	Proteobacteria	Gammaproteobacteria	Oceanospirillales	Oceanospirillaceae	
3B05	Bacteria	Proteobacteria	Gammaproteobacteria	Oceanospirillales	Oceanospirillaceae	
4H09	Bacteria	Proteobacteria	Gammaproteobacteria	Oceanospirillales	Oceanospirillaceae	
4C11	Bacteria	Proteobacteria	Gammaproteobacteria	Oceanospirillales	Oceanospirillaceae	
4A09	Bacteria	Proteobacteria	Gammaproteobacteria	Oceanospirillales	Oceanospirillaceae	
4D10	Bacteria	Proteobacteria	Gammaproteobacteria	Oceanospirillales	Oceanospirillaceae	
1F03	Bacteria	Proteobacteria	Gammaproteobacteria	Alteromonadales	Colwelliaceae	Colwellia
6E02	Bacteria	Proteobacteria	Gammaproteobacteria	Alteromonadales	Colwelliaceae	Colwellia
4B03	Bacteria	Proteobacteria	Gammaproteobacteria	Alteromonadales	Alteromonadaceae	Alteromonas
3D04	Bacteria	Proteobacteria	Gammaproteobacteria	Alteromonadales	Alteromonadaceae	Alteromonas
6C01	Bacteria	Proteobacteria	Gammaproteobacteria	Alteromonadales	Alteromonadaceae	Alteromonas
4D01	Bacteria	Proteobacteria	Gammaproteobacteria	Alteromonadales	Pseudoalteromonadaceae	Pseudoalteromonas
3D05	Bacteria	Proteobacteria	Gammaproteobacteria	Alteromonadales	Pseudoalteromonadaceae	Pseudoalteromonas
3F01	Bacteria	Proteobacteria	Gammaproteobacteria	Alteromonadales	Pseudoalteromonadaceae	Pseudoalteromonas
6C06	Bacteria	Proteobacteria	Gammaproteobacteria	Alteromonadales	Psychromonadaceae	Psychromonas
6E03	Bacteria	Proteobacteria	Gammaproteobacteria	Alteromonadales	Psychromonadaceae	Psychromonas
6H04	Bacteria	Proteobacteria	Gammaproteobacteria	Alteromonadales	Psychromonadaceae	Psychromonas
6D02	Bacteria	Proteobacteria	Gammaproteobacteria	Alteromonadales	NB-1d	
6G02	Bacteria	Proteobacteria	Gammaproteobacteria	Alteromonadales	NB-1d	
6D03	Bacteria	Proteobacteria	Gammaproteobacteria	Vibrionales	Vibrionaceae	
6E01	Bacteria	Proteobacteria	Gammaproteobacteria	Vibrionales	Vibrionaceae	
1A01	Bacteria	Proteobacteria	Gammaproteobacteria	Vibrionales	Vibrionaceae	Vibrio
3C02	Bacteria	Proteobacteria	Gammaproteobacteria	Vibrionales	Vibrionaceae	Vibrio
4B04	Bacteria	Proteobacteria	Gammaproteobacteria	Vibrionales	Vibrionaceae	Vibrio

Supplementary Figure 5 | Phylogenetic tree of phenotyped isolates. Isolate full-length 16S sequences (roughly 1,400 basepairs) were aligned against the Silva reference database. From this alignment, a maximum-likelihood tree was generated with PhyML (substitution model: GTR+gamma). Taxonomic classifications for strains (identified with the Ribosomal Protein Database) are indicated in the table. Classifications with <80% confidence as determined by RDP are not shown.

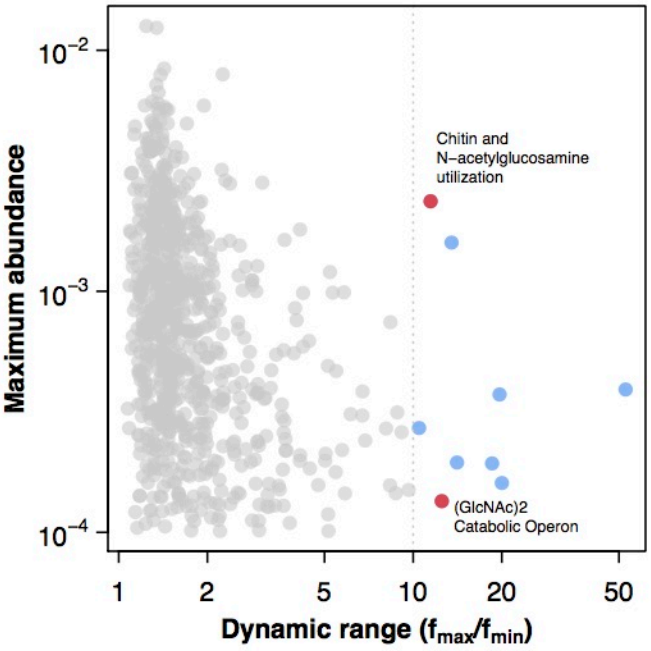

Supplementary Figure 6 | Specificity of enrichment of chitin metabolism. The maximum relative abundance reached versus the overall dynamic range for functional categories. Each point corresponds to a single functional category as annotated with MG-RAST (SEED Subsystem Level 3, similar to a KEGG pathway). Only functional categories that reached a maximum relative abundance > 10^{-4} are plotted. Functional categories for which the dynamic range (maximum abundance divided by minimum abundance) was less than 10 are plotted in gray. Those with a dynamic range greater than 10 are: $(GlcNac)_2$ Catabolic Operon, Alkanesulfonates Utilization, Atlg48360, Chitin and N-acetylglucosamine utilization, Conjugative transfer, Conjugative transposon, Bacteroidales, Ectoine biosynthesis and regulation, Heme biosynthesis orphans, Phage tail proteins 2.

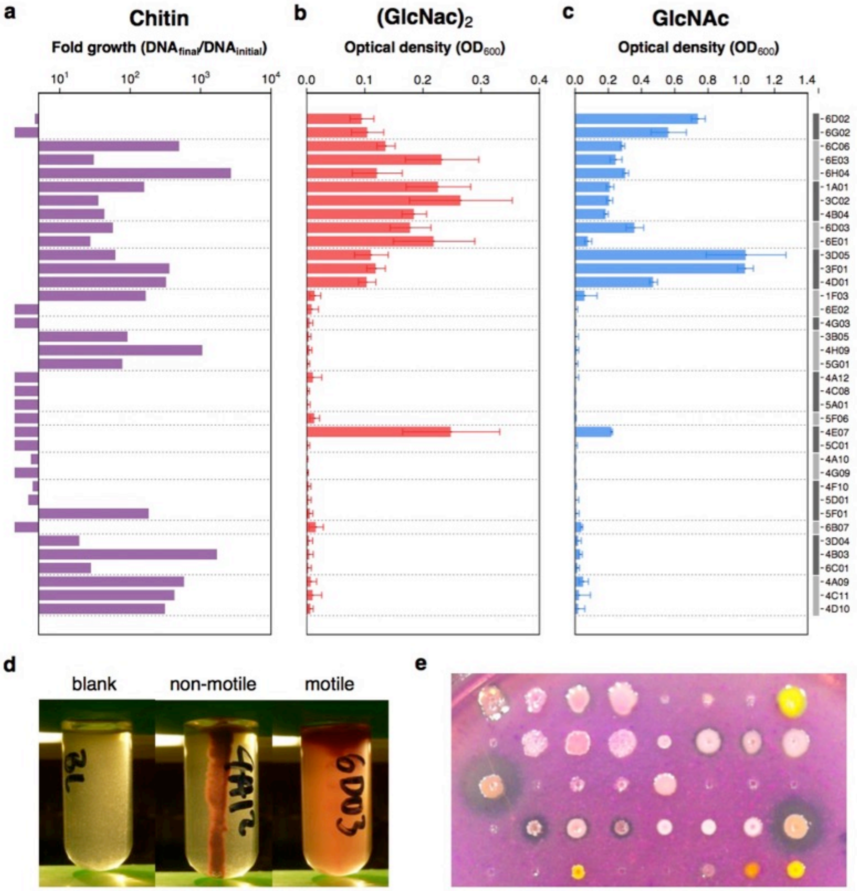

Supplementary Figure 7 | Isolate phenotypes. Phenotypes of isolate representatives of OTUs from 16S sequencing. Isolate names are indicated on the right and are grouped by the OTUs to which they correspond (light/dark gray bars). Here, OTU order corresponds to ordering in Fig. 3b. **a,** Isolate fold growth with chitin particles (1,000 particles/mL) as the sole carbon source. Culture growth was assayed by quantifying the amount of DNA present in the sample, both initially (t = 0 days) and after growth (t = 7 days). Fold growth is defined as $[DNA]_{t=7}/[DNA]_{t=0}$. Isolates for which fold growth < 5 (falling to the left of the y axis) were deemed to have no significant growth. **b,** Isolate growth with N-acetylglucosamine (GlcNAc) (0.5% w/v) as the sole carbon source. Total culture yield was assayed after 48 hours via optical density (OD_{600}). Error bars are standard deviations (n=4). **c,** Isolate growth with N',N'-diacetylchitobiose ($(GlcNAc)_2$) (0.1% w/v) as the sole carbon source. Total culture yield was assayed after 48 hours via optical density (OD_{600}). Error bars are standard deviations (n=4). **d,** Example results from agar stab assay of motility after 7 days of incubation. Left image is an un-inoculated tube (no evidence of growth). Middle image is for a non-motile strain (growth along stab line, no growth elsewhere). Right image is for a motile strain (growth throughout tube). **e,** Example from chitinase secretion assay. Each spot corresponds to the growth for an individual isolate. Zones of clearing around a colony, indicating secretion of extracellular chitinases, can be assessed visually.

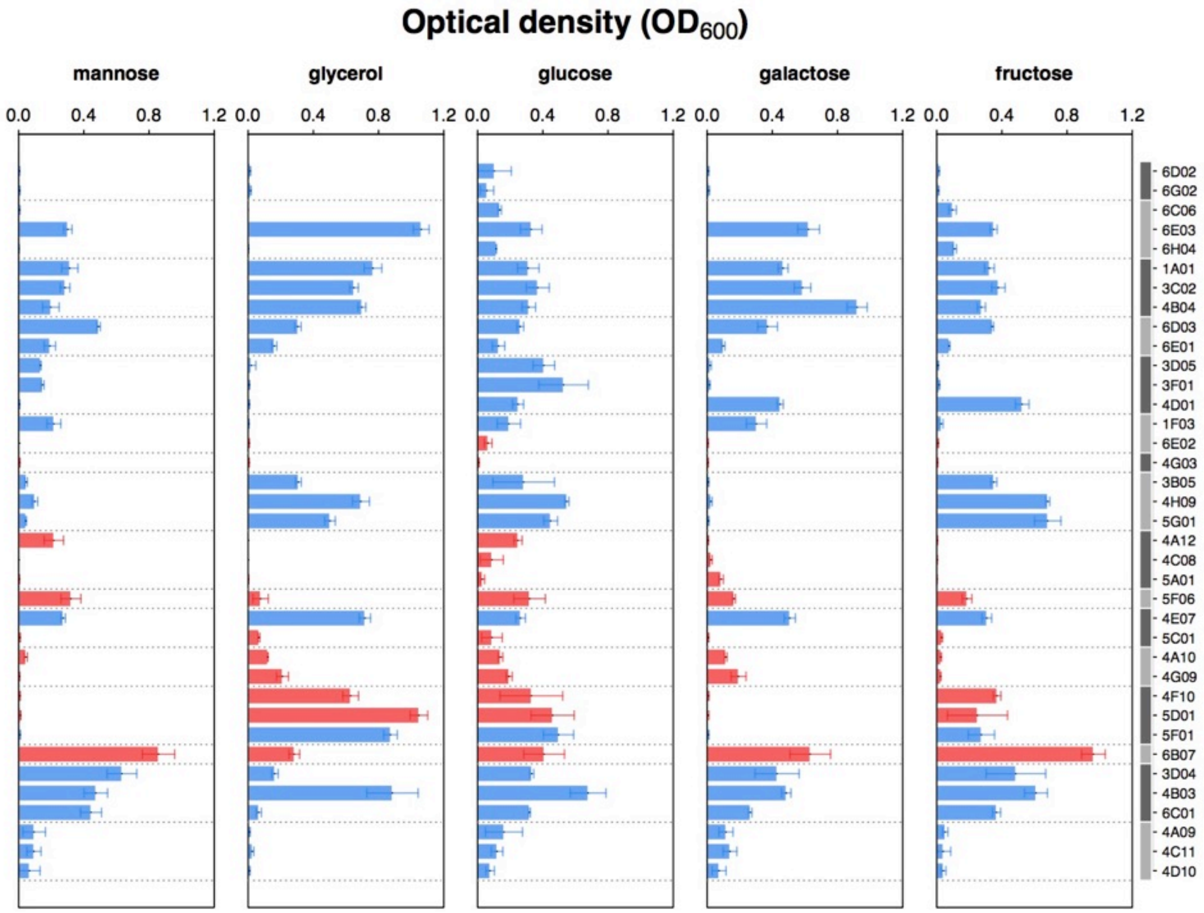

Supplementary Figure 8 | Growth of isolates with other carbon sources. Individual isolates were grown with mannose, glycerol, glucose, galactose, or fructose as the sole carbon source (0.5% w/v). Total yield of cultures was assayed after 48 hours via optical density (OD_{600}). Isolate names are indicated on the right. Isolates are grouped by the OTUs to which they correspond (light/dark gray bars), and OTU order corresponds to ordering in Fig. 3b. Data for strains that could not grow on chitin, GlcNAc, or $(GlcNAc)_2$ are indicated with red bars. Error bars are standard deviations (n=4).

Supplementary Discussion

Bacteria are the dominant particle colonizers at early stages of colonization. Seawater contains a diverse range of microbial life, including not just bacteria, but also viruses, phytoplankton, and zooplankton, among others. However, in this work, we have largely focused on the bacterial dynamics of particle colonization for the following reasons:

- *Bacteria are typically the first colonizers of naturally occurring particles and dominate at early stages.* It has been observed that, during the early phases of particle decomposition, bacterial abundance increases rapidly. Subsequently, as bacterial biofilms form and particles aggregate, microscopic bacterivores and other eukaryotes increase in abundance within particle-attached communities. This pattern has been observed for naturally occurring particles[4], as well as in simplified laboratory systems[5].
- *Empirically, bacteria were the dominant particle colonizers on the timescale of our experiment* (Supplementary Fig. 2).

Nonetheless, it is possible that non-bacterial microbes influence bacterial colonization dynamics. This includes turnover due to viral predation or eukaryotic grazers, or interkingdom signaling between phytoplankton and bacteria[6]. This will undoubtedly be an interesting subject of future work.

Alternative modes of chitin degradation. In this study, we observed a small subset of taxa that were:

- Able to grow on chitin as the sole source of carbon
- Do not secrete chitinases extracellularly
- Could not grow on GlcNAc or $(GlcNAc)_2$ as the sole source of carbon

In the canonical mode of chitin degradation, bacteria secrete chitinases into the environment. These chitinases are active extracellularly, thus allowing bacteria to degrade insoluble chitin polymers with which they do not have direct contact.

Given that the taxon subset of interest does not secrete chitinases extracellularly, they do not follow this canonical strategy. However, an alternative mode of chitin degradation has been documented in which bacteria do not release chitinases extracellularly, but instead, tether them to their surfaces. In such cases, bacteria are assumed only to degrade chitin with which they have made surface contact. It is hypothesized that these taxa are slower to degrade insoluble chitin, but are also less prone to invasion by cheaters. We have not demonstrated for this taxon subset that the chitinases are indeed surface-tethered. However, there may be interesting mechanisms (and resulting ecological dynamics) that warrant further exploration.

Even without releasing chitinases extracellularly, we might still expect taxa with surface-tethered chitinases to be able to grow on common chitinase degradation

products (GlcNAc and $(GlcNAc)_2$). However, the isolates that we collected in this study did not. We have not identified the mechanism that allows for this particular growth pattern. However, we have listed some hypothetical mechanisms below that could be tested experimentally:

- **The chitinases produced by these taxa generate larger (or different) enzymatic degradation products.** Thus, these strains are not genetically equipped to import or metabolize GlcNAc or $(GlcNAc)_2$, but may be able to metabolize other degradation products.

- **These strains consume chitin degradation products, but only in the presence of chitin.** To our knowledge, such a mechanism has not been documented. However, it is known that chitin degradation itself is dependent on both the genetic and environmental context. Thus, consumption of chitin degradation products may be similarly regulated.

- **These strains do not produce chitinases, but instead produce chitin deacetylases.** Such strains could produce acetate (and glucosamine) as enzymatic byproducts, rather than GlcNAc and $(GlcNAc)_2$. We would predict that such strains would grow well on acetate and/or glucosamine as the sole source of carbon. Notably, chitin deacetylases have been found in marine bacteria, fungi, and insects, but have largely been studied in fungal species.

Absolute abundance trajectories are robust to variability in PCR efficiency. There is significant potential for bias in amplicon sequencing, largely due to differences the efficiency of primer binding (mostly due to primer mismatches). However, these biases should not have a significant effect on our conclusions for the reasons described below.

- The primers that we use offer broad phylogenetic coverage, particularly for marine samples. According to a recent meta-analysis (Eloe-Fadrosh, et al., *Nature Microbiology*, 2016), the 515F/806R primers miss roughly 1% of bacterial sequences from seawater samples. Of sequences missed with these primers, most are from the Candidate Phylum Radiation (including Berkelbacteria, Gracilibacteria, and others), which are rarely observed in seawater. Moreover, the taxa we see in our samples (largely Bacteroidetes and Proteobacteria) are readily amplified with these primers.

- Amplification bias due to mismatches should be restricted to the first few PCR cycles (Acinas, et al., *Applied and Environmental Microbiology*, 2005). Some sequences that can be amplified with a small number of primer mismatches, albeit with reduced efficiency. However, after 3-4 cycles, the product without any mismatches to the primer will outnumber the original mismatching template by a factor of 10. Therefore, any loss of PCR efficiency due to primer mismatches to the target should be minimal.

- Our main conclusions are not sensitive to exact abundance measurements. Our key conclusions are that (a) the overall abundance fits well to a logistic curve, and (b) the temporal abundance trends are non-monotonic for some taxa and monotonic for others. These trends are robust to possible amplification biases.

Absolute abundance trajectories are robust to variability in 16S rRNA gene copy number between individual taxa. In principle, 16S rRNA gene copy number can differ substantially between bacterial taxa. However, these differences in copy number should not qualitatively affect our results for the reasons described below.

- For any given OTU, the shape of the absolute abundance curve (e.g., Fig. 2c) does not depend on the number of 16S rRNA copies per cell. Assuming that the number of 16S copies/cell for a particular OTU (n) is constant in time, we can convert values from units of 16S V4 copies/particle to cells/particle simply by dividing by n. On a logarithmic scale (like in Fig. 2c), this has the effect of shifting the entire curve down by $\log(n)$ without changing its shape. Therefore, qualitative differences in colonization dynamics (e.g., between Phase II- and Phase III-colonizers) should not be affected by possible differences in gene copy number.

- We are studying large changes in frequency compared to variability in copy number. Across all sequenced genomes, 16S rRNA gene copy number has been found to range from 1-15 copies. However, the changes in abundance on which our conclusions are based are often factors of 1,000-10,000. Thus, such changes can't be accounted for by differences in copy number alone.

Supplementary Methods

Preparation of common reagents

A. Artificial seawater

A mixture of 40 g of sea salts (Sigma #S9883-1KG) and 1 L of Milli-Q deionized water was prepared. This mixture was filtered through a 0.22-μm filter using vacuum filtration (Corning Life Sciences #CLS430517). Note that artificial seawater is a mixture of salts that does not contain sources of carbon or nitrogen.

B. Minimal medium (no carbon source)

Minimal medium, containing sources of nitrogen, phosphorus, and sulfur, but not carbon, was prepared with a protocol adapted from Tibbles and Rawling[1].

Required reagents

- NaCl (Sigma-Aldrich #S3014)
- $MgSO_4 \cdot 7H_2O$ (Sigma-Aldrich #63138)
- $MgCl_2 \cdot 6H_2O$ (Sigma-Aldrich #M2393)
- $CaCl_2 \cdot 2H_2O$ (Sigma-Aldrich #C7902)
- NH_4Cl (Sigma-Aldrich #A9434)
- Tris (1M, pH 8.0) (ThermoFisher Scientific #AM9855G)
- Disodium EDTA (0.5 M, pH 8.0) (ThermoFisher Scientific #AM9260G)
- K_2HPO_4 (Sigma-Aldrich #P3786)
- KH_2PO_4 (Sigma-Aldrich #P5655)
- $FeSO_4 \cdot 7H_2O$ (Sigma-Aldrich #F8633)
- $Na_2MoO_4 \cdot 2H_2O$ (Sigma-Aldrich #331058)
- Vitamin solution, 1000X (as previously described[2]
- Trace metals solution, 1000X (as previously described[1]

For 1 liter of minimal medium:

1. Prepare "Part I" (2X) solution.
    a. Add the following components to deionized water (final volume of 450 mL).

Component	Amount
NaCl	51.9 g
$MgSO_4 \cdot 7H_2O$	6 g
$MgCl_2 \cdot 6H_2O$	4 g
$CaCl_2 \cdot 2H_2O$	0.24 g
Tris (1M, pH 8.0)	50 mL
Na_2EDTA (0.5 M)	5.4 mL
NH_4Cl (1M)	20 mL

b. Adjust pH of solution to 7.8.
c. Add deionized water to a final volume of 500 mL.
d. Autoclave solution to sterilize.

2. Prepare "Part II" (2X) solution.

Component	Amount
K_2HPO_4	1.6 g
KH_2PO_4	0.4 g

a. Adjust pH of solution to 7.8.
b. Add deionized water to a final volume of 500 mL.
c. Autoclave solution to sterilize.

Note: For long-term storage, keep Part I and Part II in separate containers.

3. Once cooled, combine Parts I and II with the following additives:

**For 1 L of media**	
Part I (2X)	250 mL
Part II (2X)	250 mL
$FeSO_4$ solution (1000X)	1 mL
Na_2MoO_4 solution (1000X)	1 mL
Vitamins (1000X)	1 mL
Trace metals (1000X)	1 mL
Carbon source solution	?
Deionized water	to 1000 mL

Genomic DNA extractions. Before DNA extractions were performed, all samples were frozen at -80°C for purposes of long-term storage and cell lysis. Total genomic DNA was extracted from samples using a MasterPure™ DNA Purification Kit (Epicentre #MCD85201). The protocol for "fluid samples" was used with the following modifications:

a) Reagent volumes were scaled up to accommodate a 500 µL sample
b) Before isopropanol was added, glycogen (Fermentas #R0551) was added to each sample (final concentration of 0.5 µg/µL) to increase yield.
c) Following the addition of isopropanol, samples were stored at -20°C overnight to increase DNA yield.
d) DNA pellets were resuspended in 50 µL of autoclaved Milli-Q deionized water, rather than in Tris-EDTA buffer.

Quantification of total particle-attached bacteria. To quantify the total number of 16S V4 copies per particle over time, we performed quantitative PCR (qPCR) assays for each sample as described below.

Setup for qPCR reactions

Primers:
515F (GTGCCAGCMGCCGCGGTAA)
806R (GGACTACHVGGGTWTCTAAT)

PCR reaction setup:

	Volume (1 reaction)
ddH_2O	9.4 µL
5X HF Buffer	4 µL
dNTPs (10 mM)	0.4 µL
515F (10 µM)	2 µL
806R (10 µM)	2 µL
Template	2 µL
Phusion	0.2 µL
SYBR Green I (200X)	0.1 µL

"Phusion" = Phusion High-Fidelity Polymerase (New England Biolabs #M0530L). SYBR Green I working stock (at 200X) was prepared by diluting the original concentrated stock provided by the manufacturer (10,000X) with DMSO.

Cycling conditions:

Step	Temperature	Time
**Initial denaturation**	98°C	30 seconds
**Amplification (35 cycles)**	98°C	10 seconds
	50°C	60 seconds
	72°C	90 seconds
**Final extension**	72°C	10 minutes

Quantitative PCR reactions were performed with a Bio-Rad CFX96 Real-Time PCR Detection System.

Preparation of standard curve
A sample of 16S V4 amplicons (amplified from a mixture of equal volumes of genomic DNA from all timepoints) was used to prepare the standard curve. Total double-stranded DNA content was measured for this sample using a Quant-iT™ PicoGreen® dsDNA Assay Kit (Life Technologies #P7589). Standards were prepared with serial 10-fold dilutions (8 in total) from this sample. The concentration for each of these standards was assumed to be consistent with the dilution. Quantitative PCR was

performed in triplicate for each of these samples (with the protocol described above), thereby allowing a C_t value to be estimated for each standard.

In accordance with theory, a plot of the C_t value vs. log(DNA concentration) was found to be linear over a wide range of DNA concentrations, saturating at very low concentrations. Linear regression was used to obtain the equation for the best-fit line of the non-saturating data points in log-linear space. This equation was used to calculate the amount of DNA in each sample.

Limit of detection
The limit of detection for the assay was defined in the following manner:

a. Identify the most concentrated standard whose C_t is indistinguishable from the no-template controls.
b. Calculate the standard deviation of the C_t
c. LOD = $C_{t,\,neg} + 2{*}SD(C_{t,neg})$

Imaging of particle-attached communities. Samples were fixed for imaging by adding formalin at 1% by volume, and then storing the samples for 24 hours at 4°C. After this incubation period, the formalin/artificial seawater mixture was removed from particles, using a neodymium magnet (McMaster-Carr #5862K38) to separate particles from supernatant. Particles were re-suspended in PBS and stored at 4°C.

Before imaging, samples were stained with a double-stranded DNA stain (SYBR Green I Nucleic Acid Gel Stain; Life Technologies #S-7563). A working stock of the DNA stain (at 200X) was prepared in DMSO from the concentrated stock solution (10,000X). This stock was added at 0.5% v/v (final concentration was 1X in solution) to each bead sample. Beads were imaged with a Zeiss epifluorescence microscope at 40X magnification. Excitation and emission spectra of SYBR Green I are published by the manufacturers.

Calculating the effective number of species (N_{eff}) in a community. The effective number of species (N_{eff}) was calculated based upon the Shannon diversity of the community as described in Jost, 2006[3].

$$\text{Shannon Diversity} = D = -\sum_{i=1}^{n} p_i \ln p_i$$

$$\mathrm{N_{eff}} = e^{D}$$

Metagenomic sequencing of particle-attached communities. The amount of DNA present was measured with a Qubit® dsDNA HS (High Sensitivity) Assay Kit (ThermoFisher Scientific #Q32851).

For library preparation, 1 ng of each sample was used. Libraries were prepared with the Illumina Nextera XT DNA Sample Preparation Kit (Illumina # FC-131-1024) and Illumina Nextera XT DNA Sample Preparation Index Kit (Illumina # FC-131-1001). Since our libraries resulted in a wide range of fragment sizes and were at low concentrations overall, the Nextera XT library normalization protocol was not used. Instead, a double-

sided size selection was performed with Agencourt AMPure XP beads to select for DNA fragments between 300-700 basepairs. The concentration of fragments between 300-700 basepairs was assessed with an Agilent 2100 Bioanalyzer, after which samples were pooled.

Sanger sequencing of 16S rRNA for isolates. For taxonomic classification of isolates, the full 16S rRNA gene of each strain was sequenced using Sanger sequencing. To prepare DNA for sequencing, a large portion of the 16S rRNA gene was amplified by PCR under the following reaction conditions:

Primers:
27F (AGAGTTTGATCMTGGCTCAG)
1492R (TACGGYTACCTTGTTACGACTT)

PCR reaction setup:

	Volume (1 reaction)
ddH$_2$O	9.4 µL
5X HF Buffer	4 µL
dNTPs (10 mM)	0.4 µL
27F (3 µM)	2 µL
1492R (3 µM)	2 µL
Template	2 µL
Phusion	0.2 µL

"Phusion" refers to Phusion High-Fidelity Polymerase (New England Biolabs #M0530L). Instead of using genomic DNA as the template, 2 µL of a saturated culture (grown in Marine Broth 2216; Difco #279110) diluted 1:1000 in deionized water was added directly to the PCR reaction tube.

Cycling conditions:

Step	Temperature	Time
**Initial denaturation**	98°C	30 seconds
**Amplification (30 cycles)**	98°C	30 seconds
	50°C	30 seconds
	72°C	90 seconds
**Final extension**	72°C	10 minutes

Following the PCR reaction, isopropanol precipitation was used to purify the PCR products. Two Sanger sequencing reactions were performed for each of these purified PCR products, using either the forward (27F) or reverse (1492R) PCR primer as the sequencing primer (GENEWIZ, Inc.). The sequences obtained from the two reactions were merged to obtain, in the majority of cases, the fully sequenced PCR construct.

Mapping isolates to OTU sequences. Isolates were mapped at 100% identity to the OTU V4 sequences identified during 16S sequencing. Note that, while isolates mapped to a particular OTU are identical in the 16S V4 hypervariable region, they are often not identical in other regions of the 16S rRNA gene.

Supplementary references

1. Tibbles, B. J. & Rawlings, D. E. Characterization of nitrogen-fixing bacteria from a temperate saltmarsh lagoon, including isolates that produce ethane from acetylene. *Microb Ecol* **27,** 65–80 (1994).
2. Finster, K., Tanimoto, Y. & Bak, F. Fermentation of methanethiol and dimethylsulfide by a newly isolated methanogenic bacterium. *Archives of Microbiology* **157,** 425–430 (1992).
3. Jost, L. Entropy and diversity. *Oikos* **113,** 363–375 (2006).
4. Biddanda, B. A. & Pomeroy, L. R. *Microbial aggregation and degradation of phytoplankton-derived detritus in seawater. I. Microbial succession.* (Marine ecology progress series. Oldendorf, 1988).
5. Kiørboe, T., Tang, K., Grossart, H.-P. & Ploug, H. Dynamics of Microbial Communities on Marine Snow Aggregates: Colonization, Growth, Detachment, and Grazing Mortality of Attached Bacteria. *Appl. Environ. Microbiol.* **69,** 3036–3047 (2003).
6. Amin, S. A. *et al.* Interaction and signalling between a cosmopolitan phytoplankton and associated bacteria. *Nature* **522,** 98–101 (2015).
